# Ethanol extract of *Schisandrae chinensis fructus* ameliorates the extent of experimentally induced atherosclerosis in rats by increasing antioxidant capacity and improving endothelial dysfunction

**DOI:** 10.1080/13880209.2018.1523933

**Published:** 2018-12-04

**Authors:** Xiu Chen, Jiahong Cao, Yong Sun, Yaolan Dai, Jiali Zhu, Xuemei Zhang, Xiaoqin Zhao, Liwen Wang, Tingting Zhao, Yongbiao Li, Youping Liu, Guihua Wei, Tiane Zhang, Zhiyong Yan

**Affiliations:** a School of Life Science and Engineering, Southwest Jiaotong University, Chengdu, PR China;; b School of Basic Medicine, Chengdu University of Traditional Chinese Medicine, Chengdu, PR China

**Keywords:** Haematoxylin–eosin (H&E) staining, lipid lowering, oxidative stress, endothelial injury, immunohistochemistry, high-performance liquid chromatography, statins

## Abstract

**Context:***Schisandrae chinensis fructus*, the dried ripe fruit of *Schisandra chinensis* (Turcz.) Baill. (Magnoliaceae) has been used for thousands of years as a traditional Chinese herb, which can attenuate and prevent the development of cardiovascular events.

**Objective:** To evaluate the effects of the ethanol extracts from *Schisandrae chinensis fructus* fruit (EESC) on experimental atherosclerosis (AS) in rats.

**Materials and methods:** Treatment with EESC (0.35, 0.7, 1.4 g/kg/d, i.g.) and simvastatin (4 mg/kg/d, i.g.) on AS rats for 3 weeks. Sprague–Dawley rats on normal chow and under water treatment were used as control. The content of schisandrin, schisandrin A and schisandrin B in EESC was detected by HPLC. Aortic pathology changes, serum biochemical indices and nuclear factor E2-related factor 2 (Nrf-2) and heame oxygenase-1 (HO-1) expressions were measured.

**Results:** Schisandrin, schisandrin A and schisandrin B contents were 291.8, 81.46 and 279.1 mg/g of dry weight, respectively. EESC significantly reduced the aortic plaque area (76.5, 90.5 and 73.9% reduction), regulated the levels of serum lipid (*p* < 0.05), enhanced the antioxidant enzyme activities (*p* < 0.01), reduced the malondialdehyde levels (72.5, 69.3, 67.3%), and up-regulated the Nrf-2 and HO-1 expression (*p* < 0.05). Furthermore, EESC reduced the levels of oxidized-LDL and endothelin-1 and thromboxane B2 but increased that of 6-keto prostaglandin F1α (*p* < 0.05). Acute toxicity was calculated on mice to be LD_50_ > 20 g/kg.

**Conclusions:** EESC positively affects the treatment of AS *in vivo* and the findings will provide a reliable theoretical basis for developing novel therapeutics.

## Introduction

Atherosclerosis (AS) is a chronic and deadly disease caused by genetic and environmental factors worldwide (Qian et al. 2017). Clinical studies have shown that AS is the pathological basis of cardiovascular disease (Davidson 2007; Fishbein and Fishbein 2015). Abnormal lipid metabolism, endothelial dysfunction, inflammatory responses and oxidative stress occur at the early stage of AS lesions (Zhang et al. 2013). Oxidization of low density lipoprotein (LDL) in the arterial wall is critical for the development of AS plaque (Steinberg 1997; Savel et al. 2012), whereas endothelial dysfunction is the initiating factor of AS. Clinical treatment of AS includes commonly used drugs such as statins (Fu et al. 2017), fibrates (Niu et al. 2017), niacin (Li et al. 2016) and other lipid-lowering drugs; however, long-term use of lipid-lowering drugs have side effects. Therefore, exploring the safety and effectiveness of traditional Chinese medicine is a promising direction.

*Schisandrae chinensis fructus* (SCF), the dried ripe fruit of *Schisandra chinensis* (Turcz.) Baill. (Magnoliaceae), has been used for thousands of years as a traditional Chinese herb (Chinese Pharmacopeia Commission 2015; Sun et al. 2017). Clinical studies have shown that SCF can be used to protect the liver (Panossian and Wikman 2008), treat tumour (Xu et al. 2011) and act as an antioxidant (Kang et al. 2014) and anti-ageing agents. SCF combined with other herbs has been traditionally used for the prevention and treatment of cardiovascular diseases (Li et al. 1996; Panossian and Wikman 2008). *In vitro* experiments and animal experiments showed that SCF has a relaxing effect on blood vessels and inhibits platelet aggregation. The use of SCF for the treatment of cardiovascular diseases such as myocardial infarction and hypertension has recently attracted attention (Panossian and Wikman 2008; Alexander and Wang 2012; Park et al. 2012). However, the anti-AS effect of SCF extracts is not yet reported. Therefore, we attempt to investigate the effect and underlying mechanisms of EESC on a high-fat and vitamin D_3_-induced model of AS in rats, which will serve as the basis for using SCF to prevent and treat AS in the near future.

## Materials and methods

### Chemicals

Schisandrin (HPLC >98%), schisandrin A (HPLC >98%), schisandrin B (HPLC >98%) were purchased from Chengdu Pusi Biotechnology Company (Chengdu, China). Chloral hydrate was obtained from Chengdu Kelong Chemical Reagent Factory (Chengdu, China). Triglyceride (TG), low-density lipoprotein cholesterol (LDL-C), high-density lipoprotein cholesterol (HDL-C), glutathione peroxidase (GSH-PX), catalase (CAT), superoxide dismutase (SOD), malondialdehyde (MDA), oxidized low-density lipoprotein (ox-LDL), endothelin-1 (ET-1), thromboxane B_2_ (TXB_2_) and 6-keto prostaglandin F1α (6-keto-PGF_1α_) assay reagent kits were purchased from Nanjing Jiancheng Biological Engineering Company (Nanjing, China). Primary antibody (anti-HO-1, No. ab85309, diluted 1:30) was obtained from Abcam trading company (Shanghai, China). Primary antibody (anti-Nrf-2, No. bs-1074, diluted 1:100) was obtained from Beijing Boaosen Biotechnology Company (Beijing, China). The secondary antibody (No. SP-9001), diaminobenzidine (DAB) reagent kits were purchased from Beijing Zhongshan Jinqiao Biological Company (Beijing, China).

### Preparation of EESC

SCF was purchased in November 2016 from Chengdu Chinese herbal medicine market, in China, and was identified by A/Prof. Song Liangke from the School of Life Science and Engineering at Southwest Jiaotong University. The plant material (no. 20161106) was stored at the Laboratory of Life Science and Engineering, Southwest Jiaotong University (Chengdu, China). In brief, 1 kg of SCF was powdered, refluxed in 85% ethanol for 3 h, and finally filtered (plant to ethanol ratio was 1:8). The residue was refluxed for 3 h in 85% ethanol and filtered (plant to ethanol ratio was 1:6). The ethanol extract of the sample was concentrated under reduced pressure at 40 °C to obtain 382.2 g of EESC, which was stored in a refrigerator at 4 °C. The extraction rate was 38.22%.

### HPLC analysis

**Figure 1.**
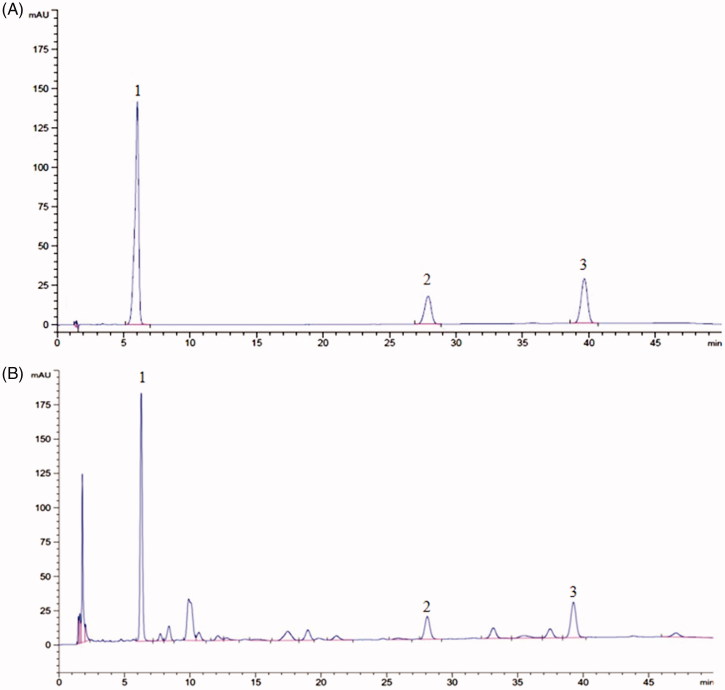
HPLC chromatogram of schisandrin, schisandrin A and schisandrin B in EESC. Schisandrin (peak 1), schisandrin A (peak 2), schisandrin B (peak 3). (A) HPLC chromatogram of schisandrin, schisandrin A, and schisandrin B. (B) HPLC chromatogram of schisandrin, schisandrin A, and schisandrin B in EESC.

HPLC analysis was performed on a Waters C18 column (150.0 mm × 4.6 mm, 5 μm) at the temperature of 30 °C. The mobile phase was methanol:water (at 0–12 min, 65:35 *v*/*v*; at 12–45 min, 75:25, *v*/*v*; at 45–50 min, 65:35, *v*/*v*) with a flow rate of 1.0 mL/min (Ni and Yang, 2012). The wavelength used to detect for schisandrin, schisandrin A and schisandrin B was set at 250 nm. EESC was dissolved in methanol and filtered through a 0.45 μm membrane filter before loading into the HPLC system. The chromatography peaks of schisandrin, schisandrin A and schisandrin B in EESC were confirmed by comparing the retention time with that of reference compounds. Results are shown in Figure 1, according to the regression equation of schisandrin, schisandrin A and schisandrin B were: *Y* = 3531.7*X*–9.8667 (*r* = 0.9999), *Y* = 2884.6*X*–3.7067 (*r* = 0.9999), *Y* = 1336.5*X*–4.2467 (*r* = 0.9999), calculated quantity of schisandrin, schisandrin A and schisandrin B of EESC were 291.8, 81.46 and 279.1 mg/g, respectively.

### Experimental animals

Sixty Sprague–Dawley (SD) rats (4 weeks old, weigh 120–140 g) and 20 Kunming (KM) mice (6 weeks old, weigh 22–24 g) of both sexes were purchased from the Dashuo Biological Technology Company in Chengdu with certificate number: SYXK (Chuan) 2015-030. The rats were fed in the SPF animal house of the Safety Evaluation Center for Natural Products in Sichuan with a 12 h light/dark cycle. The temperature was maintained at 25 °C. The bioassay was conducted according to internationally accepted guidelines for evaluating the safety and efficacy of herbal medicines (WHO 1993).

### Acute toxicity study

Acute toxicity study was performed according to the guidelines for the study of the acute toxicity of traditional Chinese medicines and natural medicines. To study any possible toxic effect, we use the limit dose of 20 g/kg for pre-experiment (*n* = 3). The animals were fasted for 12 h prior to EESC administration, and after the administration of EESC, they were monitored continuously for 2 days for any signs of toxicity, such as locomotion, behavioural, aggressiveness, reaction to stimuli, an aspect of faeces and mortality. In the experiment, 20 mice were equally divided into two groups (both sexes): drug and normal group. The drug group was administered with EESC with 0.19 g/mL concentration and 0.4 mL/10 g volume. The normal group was administered with double distilled water. The animals were observed for 14 days after administering the drugs.

### Dosing regimen

The usual dose of SCF plant drug is 3–6 g daily for an adult (Chinese Pharmacopeia Commission 2015). According to the dose–conversion relationship between human and rat (Zhu et al. 2016), the dosage of SCF was about 0.32–0.65 g/kg/d for rat. Thus, low, medium and high dosages of SCF were set as 0.35, 0.7 and 1.4 g/kg/d, respectively.

### Animals groups and model establishment

Five males and five females SD rats were randomly selected as the normal group and given a normal diet. The AS rats were fed with high-fat diet (Zhou et al. 2012), including 81.3% basal diet, 10% lard, 5% sucrose, 3% cholesterol, 0.5% sodium cholate and 0.2% propylthiouracil for 9 weeks and were injected intraperitoneally with vitamin D_3_ in the first, fourth and sixth weeks (600,000, 100,000 and 100,000 IU/kg, respectively). After the successful establishment of the model, the AS rats were randomly divided into the following five groups (five males and five females in each group): model, simvastatin (4 mg/kg/d), low-dose EESC (0.35 g/kg/d), medium-dose EESC (0.7 g/kg/d) and high-dose EESC (1.4 g/kg/d).

Drug treatments (simvastatin and EESC) were administered through the intragastric route for 3 weeks. Rats in the normal and model groups were intragastrically provided with double distilled water. The previous diet of all rats was maintained until the end of the experiment.

After the last administration, all animals were fasted for 12 h, given water freely, and then anaesthetized through the intraperitoneal injection of chloral hydrate. Blood was collected from the femoral artery of the rats and was placed into the centrifuge tube without anticoagulant. The blood was allowed to stand for 1 h at room temperature and then centrifuged for 15 min (4 °C, 4000 rpm). The supernatant was stored at –80 °C prior to detecting the serum indices.

### Histopathological examination

The chest was cut open after the collection of blood. The aorta was separated quickly and rinsed gently with saline. A 2–3 mm slice was cut from the aortic arch and fixed with freshly formulated 4% paraformaldehyde. Paraffin sections of 4 μm were prepared for haematoxylin and eosin (H&E) staining. Aortic pathology changes were observed by using a microscope (BA400Digital; Mike Audi Industrial Group Ltd., Xiamen, China). Aortic intimal plaque area and vessel wall area were measured using Image Analysis (Image-Pro Plus 6.0; United States Media Cybernetics, Rockville, MD). The percentage of the aortic plaque area was calculated as the ratio of the aortic intimal plaque area to the vessel wall area.

### Serum biochemical indices

The serum contents of TG, LDL-C, HDL-C, GSH-PX, CAT, SOD, MDA, Ox-LDL, ET-1, TXB2 and 6-keto-PGF_1α_ were measured using the reagent kits described above. The experimental procedure follows the manufacturer’s instructions using a microplate reader (BioTek Instruments, Inc., Winooski, VT, USA).

### Immunohistochemistry

The expression of nuclear factor Nrf-2 and HO-1 proteins was detected by immunohistochemical staining. The paraffin sections of aortic arch tissues cut into 4 μm thick were dewaxed, and placed in a 3% H_2_O_2_ methanol solution at room temperature for 10 min for the elimination of the endogenous peroxidase activity. Tissues were washed three times with PBS, for 5 min each time. The sections were then placed into 0.01 M citrate buffer (pH 6.0), and heated in a microwave to boiling point, after which the solution was left to cool naturally, and then washed with PBS two times for 5 min each time. Thereafter, the tissues were incubated at room temperature for 20 min with normal goat serum blocking solution and the excess liquid was gently shaken off without washing. The primary antibody (anti-HO-1, anti-Nrf-2) was added dropwise, and the tissues were then incubated at 4 °C overnight. The secondary antibody (biotin-labelled goat anti-rabbit IgG) was added dropwise, incubated at 37 °C for 30 min, and then washed with PBS three times for 5 min each time. The sections were developed in diaminobenzidine (DAB) and then washed with distilled water. Finally, the tissues were counterstained with haematoxylin, dehydrated, transformed into transparent and mounted with neutral gum. Under a trinocular microscope (BA200 Digital; Mike Audi Industrial Group, Ltd.) the positive expression of Nrf-2 and HO-1 was indicated by a brown dye. Images were collected in three fields at ×400 magnification under the microscope. Image-Pro Plus 6.0 software was used to calculate the ratio of the integral optical density to its area, and the average value was obtained.

### Statistical analysis

The experimental data were analyzed by SPSS 20.0 software (IBM Corp., Armonk, NY, USA). Data were expressed as mean ± SEM. The comparison of multiple samples was analyzed by one-way ANOVA, and the least-significant difference test was used to compare the two groups. *p* < 0.05 was considered statistically significant.

## Results

### Acute toxicity study

The results of acute toxicity study showed that all mice survived at the dose of 20 g/kg body weight. Moreover, no significant changes in breathing, defecation, impairment in food intake and yellowing or loss of hair of mice were observed. The lethal dose with 50% mortality rate (LD50) on mice is over 20 g/kg body weight. Thus, according to the dosing regimen and acute toxicity study, the three doses, namely, 0.35, 0.7 and 1.4 g/kg used in the study were free from toxic effects.

### Body weight

The body weight of rats in the normal group showed a natural growth trend over time and was higher than that in the other five groups. The weight of the rats at the 10th week was higher than that at the 9th week but decreased at 11th and 12th weeks. No significant difference was found in the weight among the different treatment groups (*p* > 0.05) (Table 1).

**Table 1.** Body weights (g).

Group	9 week	10 week	11 week	12 week
Normal	391.59 ± 42.79	408.13 ± 43.49	428.5 ± 45.69	434.72 ± 46.34
Model	358.94 ± 32.64	375.51 ± 34.16	371.71 ± 32.98	363.52 ± 30.94
Simvastatin	368.81 ± 34.92	378.23 ± 34.45	369.28 ± 32.49	364.55 ± 29.97
EESC-L	386.57 ± 38.25	398.99 ± 37.94	385.37 ± 35.44	382.41 ± 32.76
EESC-M	365.95 ± 35.49	376.24 ± 35.91	367.83 ± 33.83	360.19 ± 31.67
EESC-H	361.09 ± 36.53	364.52 ± 33.09	351.79 ± 30.25	349.86 ± 28.96

Results are expressed as mean ± SEM, *n* = 10 for each group.

Simvastatin group (4 mg/kg/d); EESC-L: low-dose EESC group (0.35 g/kg/d); EESC-M: medium-dose EESC group (0.7 g/kg/d); EESC-H: high-dose EESC group (1.4 g/kg/d).

*p* < 0.05 was considered statistically significant.

### Aortic histopathology

**Figure 2.**
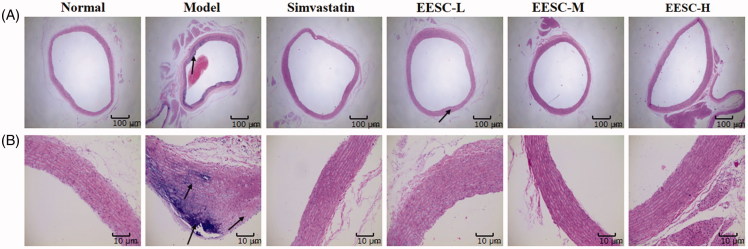
Effect of EESC on histopathological changes in rat aorta (H&E staining). (A) magnification ×40, bar: 100 μm; (B) magnification ×200, bar: 10 μm. Arrows indicate pathological changes.

The histopathological changes of the aorta were observed by H&E staining (Figure 2(A,B)). In the normal group, the arterial structure is complete, the smooth muscle cells are arranged neatly, and no obvious pathological changes were observed. In the model group, the intimal, medial and outer membrane structures are relatively complete; and plaque formation and vascular wall intima thickening were observed in the aortic tissues. The smooth muscle layer showed a focal or flake necrosis, and an unequal number of foam cells were visible in the plaque formation. Compared with the model group, the simvastatin and EESC groups showed a visible reduction of pathological changes, the vascular walls were slightly rough and thick, and AS plaque formation was not evident. Furthermore, the aortic structure is relatively complete, and the smooth muscle fibre lesions are relatively light.

**Figure 3.**
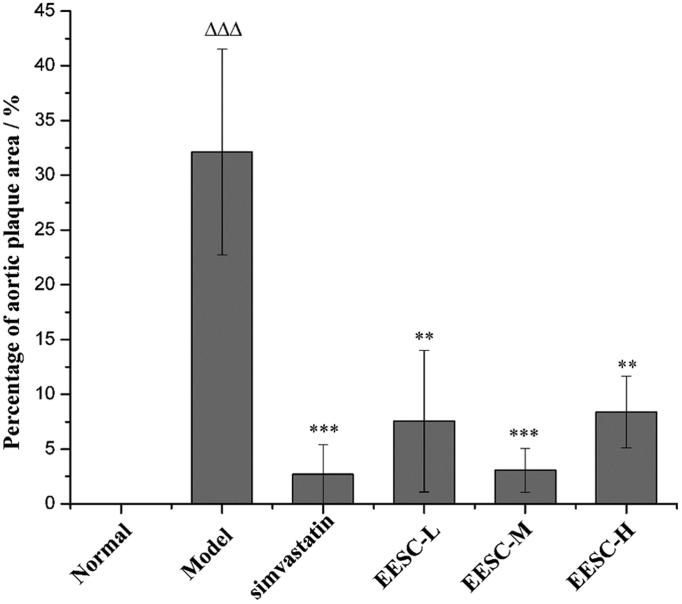
Assessment of the plaque area in the aortic arch. The proportion of aortic plaque area is expressed as the ratio of aortic intimal plaque area to the vessel wall area. Results are expressed as mean ± SEM, n = 6 for each group. *p* < 0.05 was considered statistically significant. ▵▵▵*p* < 0.001 compared with the normal group; **p < 0.01, ***p < 0.001 compared with the model group.

Compared with the normal group, the percentage of the aortic plaque area in the model group markedly increased (*p* < 0.001). The percentage of aortic plaque area (*p* < 0.01 or *p* < 0.001) significantly decreased in the simvastatin and EESC groups compared with that in the model group (Figure 3).

### Serum lipid levels

**Figure 4.**
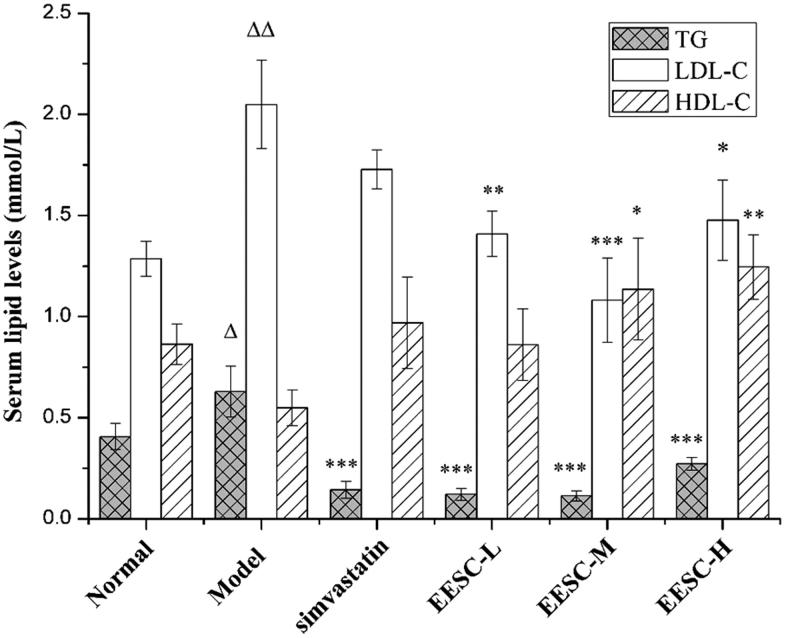
Serum lipid levels. Results are expressed as mean ± SEM, *n* = 10 for each group. *p* < 0.05 was considered statistically significant. ▵*p* < 0.05, ▵▵*p* < 0.01 compared with the normal group; *p < 0.05, **p < 0.01, ***p < 0.001 compared with the model group.

EESC can significantly regulate the serum lipid levels of AS rats. The TG and LDL-C levels in the serum significantly increased (*p* < 0.05 or *p* < 0.01) in the model group compared with those in the normal group. The TG levels (*p* < 0.001) were remarkably decreased in the drug treatment groups (simvastatin and EESC) compared with those in the model group. A markedly decreased LDL-C level was found in the medium-dose (*p* < 0.001), low-dose and high-dose groups (*p* < 0.05 or *p* < 0.01). The HDL-C level (*p* < 0.05 or *p* < 0.01) significantly increased in the medium-dose and high-dose groups (Figure 4).

### Effects of EESC on lipid peroxidation and antioxidative enzyme activities

EESC can improve the activities of GSH-PX, CAT and SOD and reduce the content of MDA. The model group had higher content of MDA (*p* < 0.01) and reduced SOD activity (*p* < 0.001) than the normal group. Compared with the model group, the MDA content in the EESC and simvastatin groups were extremely decreased (*p* < 0.01), and the activity of GSH-PX remarkably increased compared with that in the EESC groups (*p* < 0.001). The CAT activity remarkably increased in the low-dose and medium-dose (*p* < 0.001), and simvastatin groups (*p* < 0.01), and the SOD activity significantly increased in the low-dose group (*p* < 0.01) (Table 2).

**Table 2.** Effects of EESC on lipid peroxidation and antioxidative enzyme activities.

Group	MDA(nmol/mL)	GSH-PX(U/L)	CAT(U/mL)	SOD(U/mL)
Normal	5.78 ± 0.84	278.00 ± 4.09	2.32 ± 0.24	137.42 ± 4.41
Model	17.00 ± 7.00ΔΔ	257.34 ± 4.93	1.15 ± 0.21	106.77 ± 2.52ΔΔΔ
Simvastatin	5.11 ± 0.53**	271.56 ± 9.90	3.04 ± 0.86**	110.09 ± 2.80
EESC-L	4.67 ± 0.62**	328.89 ± 13.50***	4.28 ± 0.74***	120.72 ± 1.52**
EESC-M	5.22 ± 0.86**	368.89 ± 11.96***	4.40 ± 0.69***	113.62 ± 1.68
EESC-H	5.56 ± 0.62**	426.67 ± 13.58***	1.65 ± 0.46	113.13 ± 3.49

Results are expressed as mean ± SEM, *n* = 10 for each group.

Simvastatin group (4 mg/kg/d); EESC-L: low-dose EESC group (0.35 g/kg/d); EESC-M: medium-dose EESC group (0.7 g/kg/d); EESC-H: high-dose EESC group (1.4 g/kg/d).

*p* < 0.05 was considered statistically significant. ▵▵*p* < 0.01, ▵▵▵*p* < 0.001 compared with the normal group; **p < 0.01, ***p < 0.001, compared with the model group.

### Expression of Nrf-2 and HO-1 proteins in the aorta of rats

**Figure 5.**
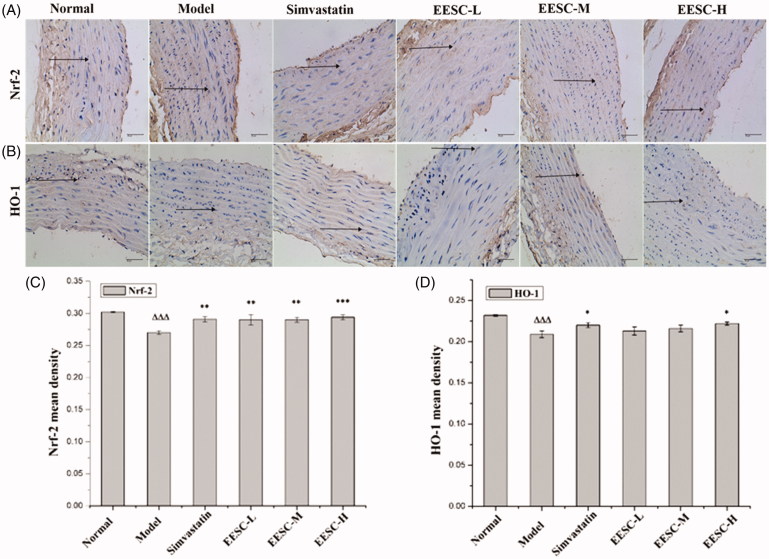
Nrf-2 and HO-1 expression were detected by immunohistochemical staining (magnification ×400, bar: 40 μm). The arrows point to positive expression of Nrf-2 and HO-1 proteins. (A) Analysis of Nrf-2 expression. (B) Analysis of HO-1 expression. (C) Nrf-2 mean density. (D) HO-1 mean density. The values are shown as mean ± SEM for six rats. ▵▵▵*p* < 0.001 compared with the normal group. *p < 0.05, **p < 0.01, ***p < 0.001 compared with the model group.

As shown in Figure 5, the background was white, the negative cells were stained blue, and the positive expression of Nrf-2 and HO-1 was dyed brown. The Nrf-2 and HO-1 protein molecules in the model group were markedly reduced compared with those in the normal group (*p* < 0.001). The Nrf-2 protein molecules significantly increased in the simvastatin, low-dose and medium-dose groups (*p* < 0.01) compared with those in the model group. A markedly increased expression of the Nrf-2 protein molecules was detected in the high-dose group (*p* < 0.001). The HO-1 protein molecules in the simvastatin and high-dose groups significantly increased (*p* < 0.05).

### Effects of EESC on vascular endothelial protection

EESC inhibited the expression of ox-LDL and ET-1 proteins but up-regulated that of 6-keto-PGF_1α_. Compared with the normal group, the model group had increased ox-LDL expression (*p* < 0.001) and TXB2 (*P* < 0.01) but decreased expression of 6-keto-PGF_1α_ (*p* < 0.05). The ox-LDL expression in the simvastatin group markedly decreased (*p* < 0.001). A significantly decreased ox-LDL expression (*p* < 0.01) was observed in the EESC groups. ET-1 expression was decreased (*p* < 0.05 or *p* < 0.01) in the simvastatin, low-dose and medium-dose groups. The TXB2 expression significantly decreased (*p* < 0.01) in the simvastatin group, and 6-keto-PGF_1α_ expression remarkably increased (*p* < 0.001) in the simvastatin and EESC groups (Table 3).

**Table 3.** Effects of EESC on vascular endothelial protection.

Group	ox-LDL(ug/mL)	ET-1(ng/L)	TXB_2_(ng/L)	6-keto-PGF_1α_(ng/L)
Normal	26.41 ± 2.81	567.28 ± 50.57	82.15 ± 8.68	122.41 ± 14.94
Model	62.61 ± 3.58ΔΔΔ	640.63 ± 86.54	162.31 ± 23.44ΔΔ	67.84 ± 11.29Δ
Simvastatin	28.92 ± 3.53***	375.98 ± 30.00**	82.66 ± 11.20**	170.03 ± 21.24***
EESC-L	43.28 ± 4.83**	382.12 ± 45.01**	145.00 ± 22.01	178.91 ± 7.69***
EESC-M	44.15 ± 4.31**	473.03 ± 54.59*	152.96 ± 17.46	244.76 ± 23.89***
EESC-H	43.07 ± 3.47**	532.43 ± 58.35	187.15 ± 14.06	210.93 ± 38.71***

Results are expressed as mean ± SEM, *n* = 10 for each group.

Simvastatin group (4 mg/kg/d); EESC-L: low-dose EESC group (0.35 g/kg/d); EESC-M: medium-dose EESC group (0.7 g/kg/d); EESC-H: high-dose EESC group (1.4 g/kg/d).

*p* < 0.05 was considered statistically significant. Δ*p* < 0.05, ΔΔ*p* < 0.01, ΔΔΔ*p* < 0.001 compared with the normal group; *p < 0.05, **p < 0.01, ***p < 0.001 compared with the model group.

## Discussion

Successful establishment of the AS models is critical for this study. The AS model was established through the intraperitoneal injection of VD_3_ and high-fat diet for 9 weeks (Zhou et al. 2011). The AS rats showed hyperlipidaemia, oxidative stress and endothelial dysfunction, all of which are indicative of the suitability of the model to be used in observing the effects of EESC on experimental AS in rats.

Simvastatin is a 3-hydroxy-3-methylglutaryl coenzyme A (HMG-CoA) reductase inhibitor (statin) that can effectively inhibit the synthesis of cholesterol (Kong et al. 2008). Lipids including TG, cholesterol (TC), cholesterol esters (CE), phospholipids and glycolipids are capable of binding to apolipoproteins to form lipoproteins, which are the predominant form of blood lipids in the body. Very low-density lipoprotein (VLDL) is the main form transporting liver-synthesized TG into the blood circulation (Wei et al. 2016). After treatment with simvastatin, cholesterol synthesis in hepatocytes decreased and the synthesis and release of VLDL were blocked, and TG level into the blood is reduced; at the same time, the expression of LDL receptor on the surface of hepatocytes is increased by negative feedback regulation, resulting in the uptake and utilization of large amounts of LDL in the blood, and the decrease of TC, LDL-C and VLDL-C levels in the blood.

In the experiment, the body weight of rats in high-fat diet group was slightly lower than that in the normal diet group, which may be due to the bitter taste of propylthiouracil, thus affecting the taste of feed, which has been reported in other studies (Ling et al. 2016). Considering that AS occurs in both men and women, the study selected rats of both sexes. The results showed that the EESC had a better effect on atherosclerotic rats (both sex). Pathological results showed that EESC could reduce the percentage of aortic plaque area and degree of smooth muscle fibre lesion, confirming that EESC can improve AS lesion.

The serum levels of TG and LDL-C significantly increased in the model group compared with those in the normal group. High levels of LDL-C can precipitate in the vascular wall, leading to the formation of plaques and eventually AS lesions (Napoli et al. 1997). The medium-dose and high-dose groups showed a significant reduction in serum levels of TG and LDL-C and an increase in serum level of HDL-C, which consequently lead to few AS plaques in these two groups. This finding suggested that EESC can prevent AS development.

Oxidative stress theory is one of the mechanisms of AS development (Collins et al. 2009). The stable biomarkers produced in the body are usually measured as a result of oxidation (Harrison et al. 2003). The biomarkers for AS include 8-hydroxy-2′-deoxyguanosine (8-OHdG; markers of oxidative DNA damage) (Lin et al. 2004), MDA (a marker of lipid peroxides) (Morrow 2005), carboxymethyl lysine and glutarin (markers of glycosylation), nitrotyrosine (markers of nitric oxide) (Tsukahara 2007; Stephens et al. 2009), SOD, GSH-PX, CAT, heame oxygenase, thioxygenin and oxyphosphatase (Durrington et al. 2001; Yamawaki et al. 2003; Tsukahara 2007). Based on the oxidative stress theory, the SOD, GSH-PX and CAT activities in the rat serum were detected by biochemical method because the activity of these enzymes could reflect the ability of the body to resist oxidation. MDA is the product of the degradation of body lipid peroxidation and can reflect the degree of oxidative stress in rats. Nrf-2 is an important regulatory factor for endogenous antioxidant stress, has a regulation effect on the endogenous antioxidant defence system, and can counteract oxidative stress induced by reactive oxygen. The Nrf-2/ARE pathway is an important antioxidant pathway (Jeong et al. 2006; Chapple et al. 2012), and the protective enzymes of GSH-PX, CAT, SOD and HO-1 are the classic target genes that directly reflect the rate and intensity of lipid peroxidation. The results showed that the expression of Nrf-2 and HO-1 proteins in the model group was significantly lower than that in the normal group due to the damage of Nrf-2 nuclear transposition. After 3 weeks of administration, the Nrf-2 expression significantly increased, indicating that the Nrf-2 nuclear translocation improved, and its corresponding target antioxidant enzyme activity and HO-1 protein expression increased. However, HO-1 expression only showed significant differences in the high dose group. We believe that it may be that the chemical composition of the EESC is complex, and different doses of EESC have different sensitivities to certain indicators. Studies have shown that the dose–effect relationship of natural drugs is not a simple linear relationship, and different doses of drugs may play different roles depending on the content of their components (Fan et al. 2009). In addition, more studies need to be conducted to confirm the expression of HO-1 and Nrf-2. Nrf-2 regulates a group of phase II antioxidant enzymes, and we need to test more Nrf-2 targeting proteins in future studies to demonstrate the role of Nrf-2 signalling pathway in the treatment of AS.

Ox-LDL is a key molecule for AS development (Montecucco and Mach 2009). ET-1, TXB_2_ and 6-keto-PGF_1α_ levels were related with endothelial dysfunction, which is an early marker of AS (Félétou and Vanhoutte 2006). Compared with the normal group, the model group had a reduced level of 6-keto-PGF_1α_ and elevated levels of ox-LDL, ET-1 and TXB_2_. After treatment with EESC, the ox-LDL and ET-1 levels were reduced in the EESC groups. The low-dose and medium-dose groups showed a reduced level of TXB_2_, whereas and increased levels of 6-keto-PGF_1α_. These results suggested that EESC administration is beneficial for AS rats by protecting the endothelium.

## Conclusions

The experimental results showed that EESC positively affects the treatment of AS by regulating the abnormal lipid metabolism, enhancing the antioxidative activities and improving the endothelial dysfunction of atherosclerotic rats. The findings in this study will provide a reliable theoretical basis for the development of SCF into new anti-atherosclerosis drugs.
